# Supplementary material for: Photonic hyperthermia of malignant peripheral nerve sheath tumors at the third near-infrared biowindow
Source: eLife. 2022 Sep 16;11:e75473. doi: 10.7554/eLife.75473 (PMC9553212; doi:10.7554/eLife.75473)
Supplement: Supplementary file 1. [file elife-75473-supp1.doc]

**Table S1.** Laser parameters (test after preheating for 5 minutes)

| Optical parameters | | |
| --- | --- | --- |
| 1.1 | Laser fiber output power | 1595mW |
| 1.2 | Wavelength (1650+/-20nm) | 1645.2nm |
| 1.3 | Preheat time | <5 minutes |
| 1.4 | Optical power stability | <3% / 2 hours |
| Optical properties | | |
| 2.1 | optical interface | SMA |
| 2.2 | Optical fiber specifications | 600um/NA:0.22/SP6.0 |
| 2.3 | Fiber length | 1.5 meter |
| Working conditions | | |
| 3.1 | Laser operating temperature range: (case temperature) | 0℃~40℃ |
| 3.2 | Power supply model | 260 case |
| 3.3 | Operating Voltage | 90-240VAC |
