# Supplementary material for: Photonic hyperthermia of malignant peripheral nerve sheath tumors at the third near-infrared biowindow
Source: eLife. 2022 Sep 16;11:e75473. doi: 10.7554/eLife.75473 (PMC9553212; doi:10.7554/eLife.75473)
Supplement: Supplementary file 2. [file elife-75473-supp2.doc]

**Table S2. Top 10 upregulated genes related to hsa04141**

| **mRNA id** | **gene id** | **gene name** | **gene location** | **log FC of 0.5 W cm^-2^** | **q value of 0.5 W cm^-2^** | **log FC of 1 W cm^-2^** | **q value of 1 W cm^-2^** |
| --- | --- | --- | --- | --- | --- | --- | --- |
| **ENST00000522855** | ENSG00000113558 | SKP1 | 5:134157572-134176950 | 16.73 | 3.21E-59 | 17.52 | 1.06E-64 |
| **ENST00000439918** | ENSG00000185624 | P4HB | 17:81843994-81860560 | 16.83 | 6.94E-60 | 17.51 | 1.18E-64 |
| **ENST00000531917** | ENSG00000163479 | SSR2 | 1:156011810-156020789 | 13.22 | 2.77E-34 | 11.43 | 5.19E-22 |
| **ENST00000370087** | ENSG00000180879 | SSR4 | X:153794191-153798498 | 12.42 | 1.02E-28 | 11.36 | 1.51E-21 |
| **ENST00000375651** | ENSG00000204389 | HSPA1A | 6:31815464-31817946 | 5.88 | 5.14E-28 | 6.31 | 3.78E-31 |
| **ENST00000374491** | ENSG00000088298 | EDEM2 | 20:35115364-35147319 | 4.47 | 2.16E-19 | 5.21 | 2.20E-24 |
| **ENST00000356638** | ENSG00000089597 | GANAB | 11:62624829-62646613 | 2.85 | 5.68E-10 | 3.03 | 5.56E-11 |
| **ENST00000404241** | ENSG00000128272 | ATF4 | 22:39519695-39522683 | 2.29 | 1.22E-06 | 2.34 | 6.45E-07 |
| **ENST00000377475** | ENSG00000101310 | SEC23B | 20:18507482-18561415 | 2.28 | 5.74E-07 | 1.91 | 2.79E-05 |
| **ENST00000375203** | ENSG00000213676 | ATF6B | 6:32115264-32128246 | 1.38 | 3.84E-03 | 1.84 | 7.77E-05 |
