# Supplementary material for: Photonic hyperthermia of malignant peripheral nerve sheath tumors at the third near-infrared biowindow
Source: eLife. 2022 Sep 16;11:e75473. doi: 10.7554/eLife.75473 (PMC9553212; doi:10.7554/eLife.75473)
Supplement: Supplementary file 3. [file elife-75473-supp3.doc]

**Table S3. Top 10 upregulated genes related to hsa04120**

| **mRNA id** | **gene id** | **gene name** | **gene location** | **log FC of 0.5 W cm^-2^** | **q value of 0.5 W cm^-2^** | **log FC of 1 W cm^-2^** | **q value of 1 W cm^-2^** |
| --- | --- | --- | --- | --- | --- | --- | --- |
| **ENST00000424381** | ENSG00000170142 | UBE2E1 | 3:23810443-23891314 | 18.76 | 1.85E-73 | 19.21 | 1.53E-76 |
| **ENST00000522855** | ENSG00000113558 | SKP1 | 5:134157572-134176950 | 16.73 | 3.21E-59 | 17.52 | 1.06E-64 |
| **ENST00000445950** | ENSG00000078140 | UBE2K | 4:39698176-39779983 | 16.66 | 1.10E-58 | 16.07 | 2.12E-54 |
| **ENST00000523815** | ENSG00000154582 | ELOC | 8:73946534-73971908 | 15.16 | 5.33E-48 | 16.03 | 4.13E-54 |
| **ENST00000512194** | ENSG00000138641 | HERC3 | 4:88670139-88687299 | 13.25 | 1.71E-34 | 12.60 | 7.63E-30 |
| **ENST00000352551** | ENSG00000175063 | UBE2C | 20:45812674-45816957 | 11.93 | 1.91E-25 | 12.41 | 1.57E-28 |
| **ENST00000415609** | ENSG00000144744 | UBA3 | 3:69054730-69080381 | 7.97 | 1.10E-29 | 7.81 | 4.17E-29 |
| **ENST00000450136** | ENSG00000119401 | TRIM32 | 9:116687302-116701298 | 5.24 | 4.96E-23 | 5.26 | 2.85E-23 |
| **ENST00000588994** | ENSG00000102858 | MGRN1 | 16:4624961-4688908 | 1.64 | 4.22E-04 | 3.50 | 1.27E-13 |
| **ENST00000270225** | ENSG00000142230 | SAE1 | 19:47130863-47210191 | 3.76 | 2.14E-15 | 3.13 | 1.44E-11 |
